# Stem Cell Factor-Inducible MITF-M Expression in Therapeutics for Acquired Skin Hyperpigmentation

**DOI:** 10.7150/thno.39066

**Published:** 2020-01-01

**Authors:** Cheong-Yong Yun, Eunmiri Roh, Song-Hee Kim, Jinhe Han, Jiyeon Lee, Da-Eun Jung, Ga Hyeon Kim, Sang-Hun Jung, Won-Jea Cho, Sang-Bae Han, Youngsoo Kim

**Affiliations:** 1 College of Pharmacy, Chungbuk National University, Cheongju 28160, Korea.; 2 College of Pharmacy, Chonnam National University, Gwangju 61186, Korea.; 3 College of Pharmacy, Chungnam National University, Daejeon 34134, Korea.

**Keywords:** Stem cell factor, KIT, MITF-M activity, epidermal melanocyte, skin pigmentation, chemical inhibition

## Abstract

**Rationale:** Microphthalmia-associated transcription factor M (MITF-M) plays important roles in the pigment production, differentiation and survival of melanocytes. Stem cell factor (SCF) and its receptor KIT stimulate MITF-M activity via phosphorylation at the post-translation level. However, the phosphorylation shortens half-life of MITF-M protein over the course of minutes. Here, we investigated novel hypotheses of (i) whether SCF/KIT can regulate MITF-M activity through gene expression as the alternative process, and (ii) whether chemical inhibition of KIT activity can mitigate the acquired pigmentation in skin by targeting the expression of MITF-M.

**Methods:** We employed melanocyte cultures *in vitro* and pigmented skin samples *in vivo*, and applied immunoblotting, RT-PCR, siRNA-based gene knockdown and confocal microscopy.

**Results:** The protein and mRNA levels of MITF-M in epidermal melanocytes and the promoter activity of MITF-M in B16-F0 melanoma cells demonstrated that SCF/KIT could trigger the expression of MITF-M *de novo*, following the phosphorylation-dependent proteolysis of pre-existing MITF-M protein. SCF/KIT regulated the transcription abilities of cAMP-responsive element-binding protein (CREB), CREB-regulated co-activator 1 (CRTC1) and SRY-related HMG-box 10 (SOX10) but not β-catenin at the MITF-M promoter. Meanwhile, chemical inhibition of KIT activity abolished SCF-induced melanin production in epidermal melanocyte cultures, as well as protected the skin from UV-B-induced hyperpigmentation in HRM2 mice or brownish guinea pigs, in which it down-regulated the expression of MITF-M *de novo* at the promoter level.

**Conclusion:** We propose the targeting of SCF/KIT-inducible MITF-M expression as a strategy in the therapeutics for acquired pigmentary disorders.

## Introduction

Acquired pigmentary disorders, such as melasma, freckles and senile lentigo, are resulted from the imbalanced production and aberrant distribution of heavily pigmented melanosomes in the skin 1, 2. The melanogenic process synthesizes melanin pigments, blackish-brown eumelanin and yellowish-red pheomelanin, in melanosomes of melanocytes and transfers the pigmented melanosomes to keratinocytes at the overlaying epidermis 3, 4. Tyrosinase (TYR) catalyzes the biosynthetic pathways of eumelanin and pheomelanin, while dopachrome tautomerase (DCT) and TYR-related protein 1 (TRP-1) are indispensable in the production of eumelanin but not of pheomelanin 4, 5. Gene expression of TYR, DCT or TRP-1 is regulated by MITF-M, a master transcription factor in the pigment production, differentiation and survival of melanocytes 6, 7, 8. Congenital defects in MITF-M activity gives rise to a disease called Waardenburg syndrome II in humans or a profound loss of pigmented cells in mice 9, 10.

SCF interacts with its receptor KIT in the skin, which influences the onset of melanogenic process 11. Treatment with KIT-neutralizing antibody inhibits UV-B-induced pigmentation in the skin of guinea pigs 12, and the production of soluble KIT from membrane-bound status abolished melanin content in SCF-activated human melanocytes 13. SCF expression is prevalently increased in the acquired pigmentary disorders 14. Congenital loss-of-function in KIT has been observed in humans, causing Piebaldism that is a disorder of melanocyte development 10, 15. SCF stimulates the intrinsic kinase activity of KIT, leading to autophosphorylation at specific Tyr residues in the cytoplasmic domain 16, 17. The phosphorylated Tyr residues in KIT recruit adaptor proteins such as Grb2, and transmit several signal cascades through Src family kinase, phosphatidylinositol 3'-kinase (PI3K) or mitogen-activated protein kinase (MAPK) 16, 17. In particular, the phosphorylation sites of KIT at Tyr-568 and -570 are important in the pigment production, since mice with targeted mutations of the two Tyr residues to Phe show a coat color change to completely white from black 18.

However, signaling pathways of SCF/KIT that link to MITF-M activity in the melanogenic process are not clarified yet. The activation of MITF-M by SCF/KIT has been claimed to occur at the post-translation level. SCF/KIT stimulates the MAPK pathway, in which extracellular signal-regulated kinase (ERK) and p90 ribosomal S6 kinase 1 (RSK-1) phosphorylate MITF-M at Ser-73 and Ser-409, respectively 19, 20. The dual phosphorylation enhances the transcriptional activity of MITF-M via recruiting the CBP/p300 co-activator 21, 22, but shortens half-life of the protein as a consequence of ubiquitin-dependent proteolysis 19. Recently, chemical inhibition of ERK or the mitogen and stress-activated protein kinase 1 (MSK1) was found to suppress the mRNA and protein levels of MITF-M or TYR in SCF-activated human melanocytes 23, 24.

In the current study, we elucidated that SCF/KIT can regulate MITF-M activity through gene expression at the promoter level, and that chemical inhibition of KIT activity can mitigate the MITF-M expression leading to pigment production in SCF-activated epidermal melanocyte *in vitro* or UV-B-exposed skin *in vivo*.

## Results

### SCF/KIT stimulated MITF-M activity through gene expression

**Figure 1:**
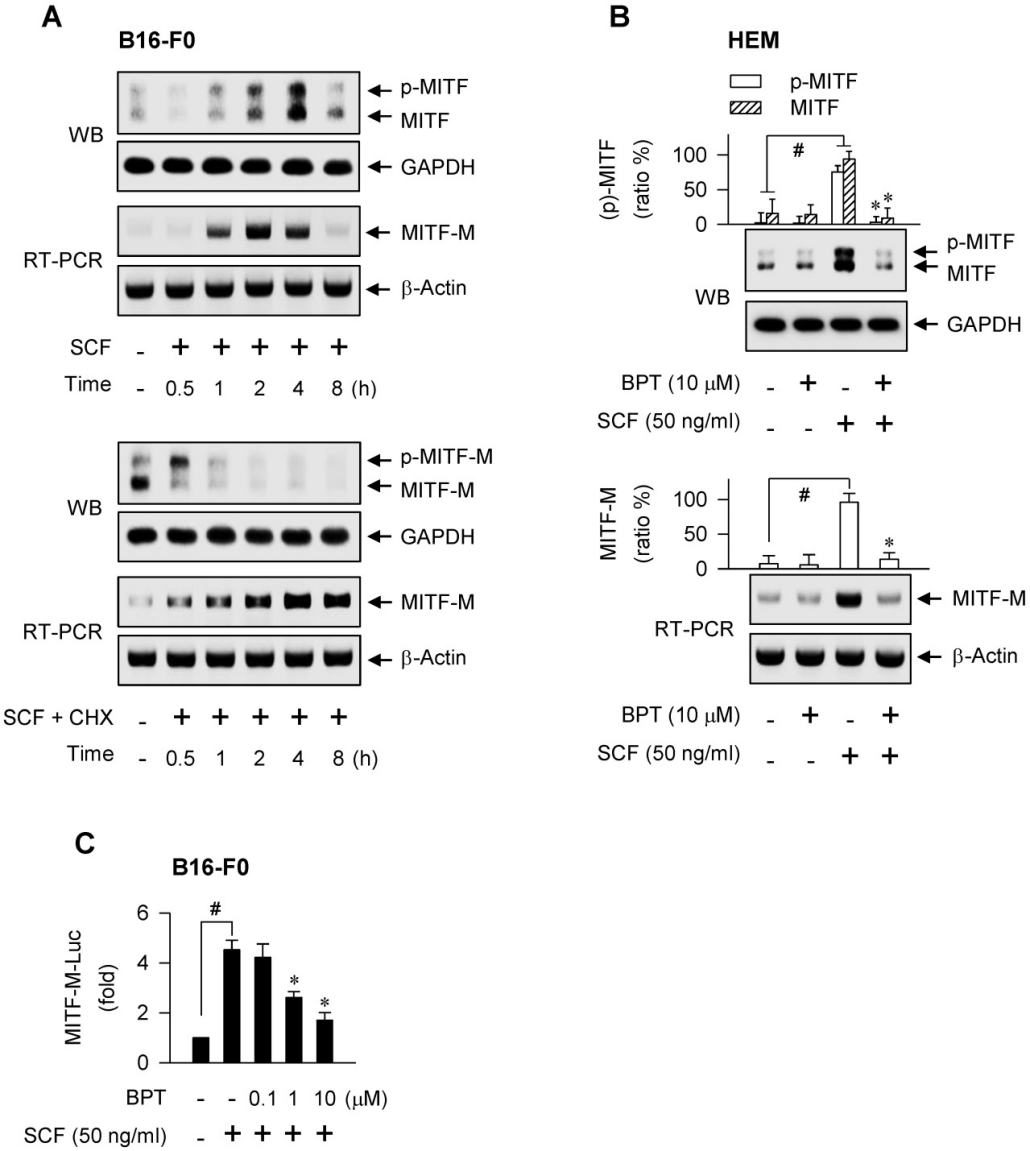
** SCF/KIT-induced MITF-M expression.** Western blot analysis (WB) and RT-PCR analysis of MITF-M. (A) B16-F0 cells were stimulated with SCF (50 ng/ml) in the absence or presence of cycloheximide (CHX, 50 μM). (B) HEM cells were pretreated with BPT for 2 h and stimulated with SCF for 4 h (WB) or 2 h (RT-PCR) in the presence of BPT. (C) Luciferase reporter analysis on the promoter activity of MITF-M. B16-F0 cells harboring MITF-M-Luc reporter construct were stimulated with SCF for 20 h in the presence of BPT. Data are mean ± SEM. ^#^*P* < 0.05 vs. medium alone. *P < 0.05 vs. SCF alone.

The observation that SCF/KIT can regulate the quantity of MITF-M through gene expression was first made in melanocyte cultures. Western blot analysis (WB) revealed two MITF-M species with the electrophoretic mobility corresponding to molecular masses 54 kDa and 60 kDa in B16-F0 and human epidermal melanocyte (HEM) cells (Figure 1A-B). The upper band has been assigned as a shift of the lower band due to phosphorylation 19, 20, and melanocyte-specific MITF-M isoform consists of 419 amino acid residues near the molecular mass 54 kDa 6, 25. Stimulation of KIT with SCF decreased the protein levels of pre-existing MITF-M over the course of minutes, and then up-regulated the expression of MITF-M *de novo* with maximal amount of protein at 4 h and mRNA at 2 h in B16-F0 cells (Figure 1A, upper). To clarify the fate of pre-existing MITF-M in response to SCF/KIT, *de novo* protein synthesis was inhibited by incubation with cycloheximide. SCF in the presence of cycloheximide shifted the electrophoretic mobility of pre-existing MITF-M protein via phosphorylation, erased the phosphor (p)-MITF-M via proteolysis, and then increased the mRNA levels of MITF-M *de novo* (Figure 1A, lower). Moreover, SCF up-regulated the protein and mRNA levels of MITF-M in HEM cells (Figure 1B). To clarify the transcriptional regulation of MITF-M, B16-F0 cells were transfected with MITF-M-Luc, a construct encoding the promoter region (-2200/+95) of MITF-M fused with the luciferase reporter. SCF markedly stimulated the luciferase activity, reporting the promoter activity of MITF-M (Figure 1C). The results indicate that SCF/KIT could control MITF-M activity through gene expression at the promoter level, following the phosphorylation-dependent proteolysis of pre-existing MITF-M protein.

**Figure 2:**
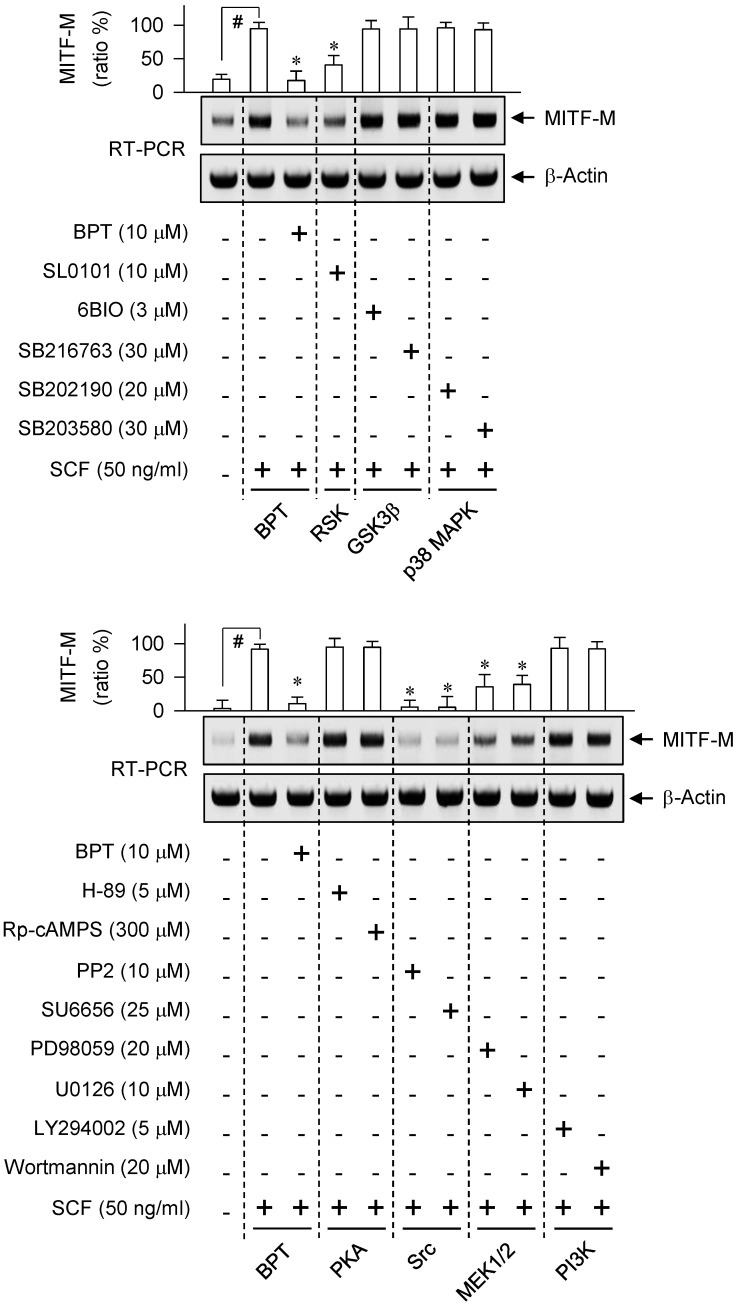
** Effect of kinase inhibitor on SCF/KIT-induced mRNA levels of MITF-M.** RT-PCR analysis of MITF-M. B16-F0 cells were pretreated with kinase inhibitor for 2 h and stimulated with SCF for another 2 h in the presence of kinase inhibitor. Data are mean ± SEM. ^#^*P* < 0.05 vs. medium alone. *P < 0.05 vs. SCF alone.

We next asked which kinase pathway could undertake SCF-induced MITF-M expression. An RSK inhibitor (SL0101), Src inhibitors (PP2, SU6656) or MEK1/2 inhibitors (PD98059, U0126) suppressed SCF-induced mRNA levels of MITF-M, while GSK3β inhibitors (6BIO, SB216763), p38 MAPK inhibitors (SB202190, SB203580), PKA inhibitors (H-89, Rp-cAMPS) and PI3K inhibitors (LY294002, wortmannin) had no significant effects (Figure 2). siRNA-based gene knockdown of Grb2 also ablated SCF-induced mRNA levels of MITF-M (Figure S1A).

**Figure 3:**
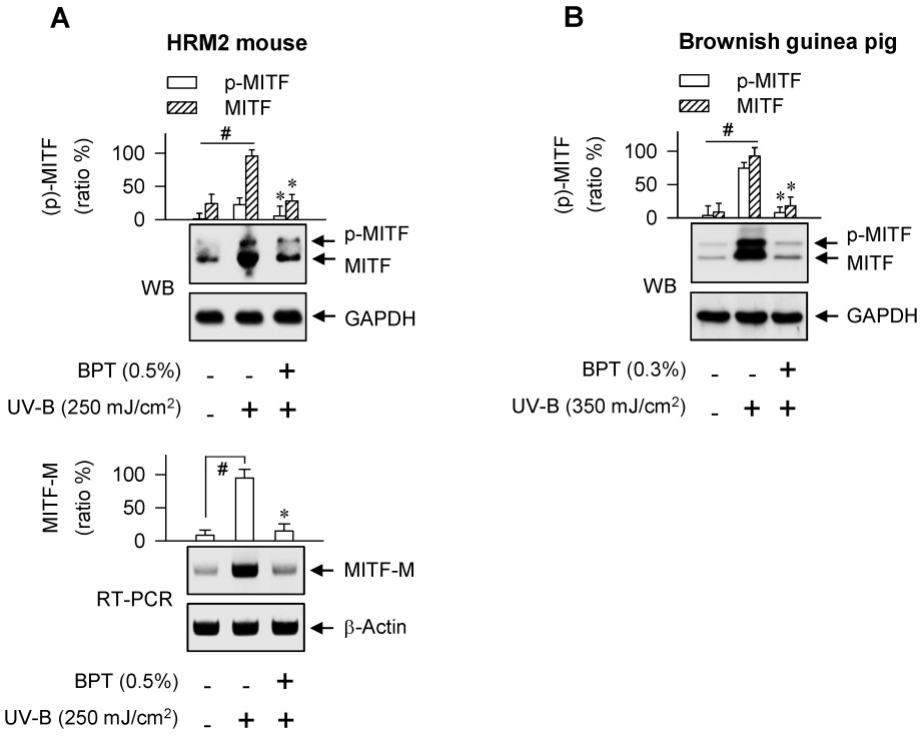
** UV-B-induced MITF-M expression.** Western blot analysis (WB) and RT-PCR analysis of MITF-M. The dorsal skin of HRM2 mice (A) or brownish guinea pigs (B) was irradiated with UV-B and treated topically with BPT as shown in Figure S1D-E. Data are mean ± SEM. ^#^*P* < 0.05 vs. normal skin. *P < 0.05 vs. UV-B alone.

Benzyl pyrimidine thione (BPT, Figure S1B) inhibits melanin production in B16-F0 cells with decrease in its efficacy when the moiety of tetrahydropyrimidine thione is replaced by imidazolidine thione or cyclic urea 26. Here, BPT suppressed the protein and mRNA levels of MITF-M in SCF-activated HEM and B16-F0 cells, as did ISCK03 and imatinib (Figure 1B; Figure S1C), and inhibited the promoter activity of MITF-M (Figure 1C). ISCK03 prevents UV-B-induced skin pigmentation in guinea pigs by attenuation of SCF/KIT signaling 27. Imatinib, an anti-leukemia drug targeting the BCR-ABL fusion protein, reduces SCF-induced melanin content in human melanocytes 28. To understand whether BPT can regulate the expression of MITF-M *in vivo*, the dorsal skin of HRM2 mice or brownish guinea pigs was irradiated with UV-B and treated topically with BPT (Figure S1D-E), mimic models for acquired hyperpigmentation in the skin. Topical BPT down-regulated the protein and mRNA levels of MITF-M in UV-B-exposed and pigmented skin (Figure 3A-B). The results indicate that BPT could inhibit MITF-M activity through gene expression in the epidermal melanocyte of UV-B-exposed skin.

### Transcription factors that switched on the MITF-M promoter in response to SCF/KIT

**Figure 4:**
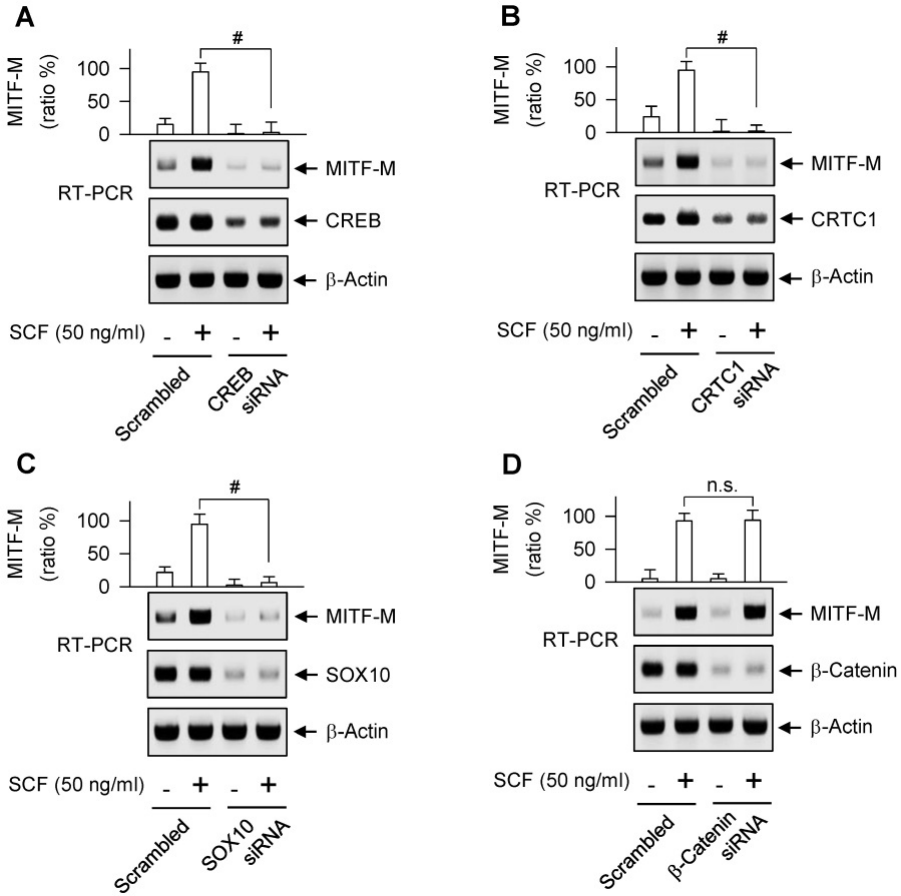
** Gene knockdown of transcription factor acting on the MITF-M promoter.** RT-PCR analysis of MITF-M. B16-F0 cells were transfected with siRNA against CREB (A), CRTC1 (B), SOX10 (C) or β-catenin (D) for 48 h and stimulated with SCF for 2 h. Data are mean ± SEM. ^#^*P* < 0.05 vs. scrambled siRNA. Abbreviation; n.s., not significant.

**Figure 5:**
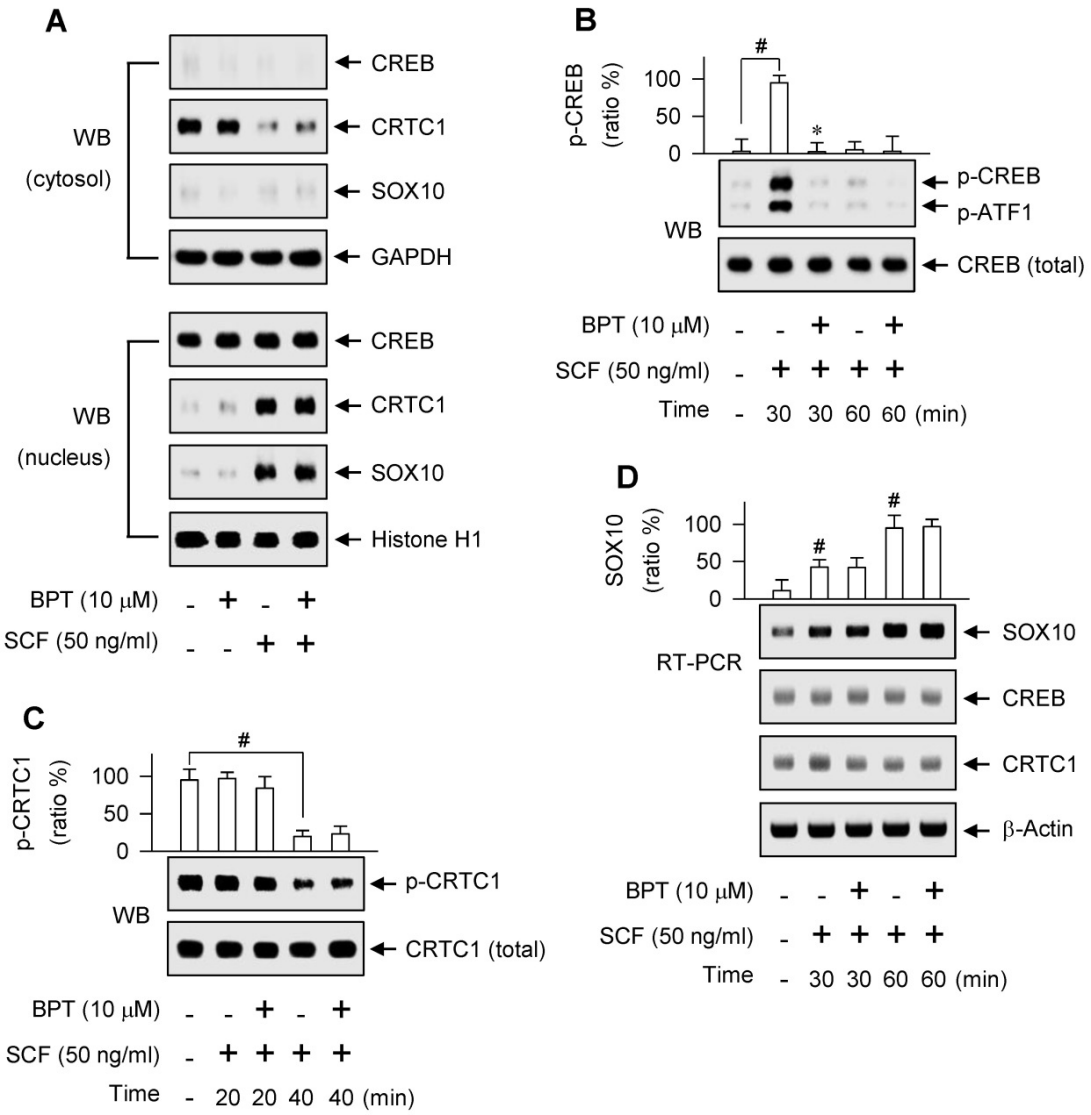
** Nuclear-cytoplasmic shuttling of CREB, CRTC1 or SOX10.** (A) Western blot analysis (WB) of CREB, CRTC1 or SOX10. B16-F0 cells were pretreated with BPT for 2 h and stimulated with SCF for 1 h in the presence of BPT. Cell extracts were partitioned between the cytosol and the nucleus. (B, C) WB on the phosphorylation of CREB or the dephosphorylation of CRTC1. B16-F0 cells were pretreated with BPT for 2 h and stimulated with SCF for indicated time points in the presence of BPT. (D) RT-PCR analysis on the induction of SOX10. B16-F0 cells were pretreated with BPT for 2 h and stimulated with SCF for indicated time points in the presence of BPT. Data are mean ± SEM. ^#^*P* < 0.05 vs. medium alone. *P < 0.05 vs. SCF alone.

**Figure 6:**
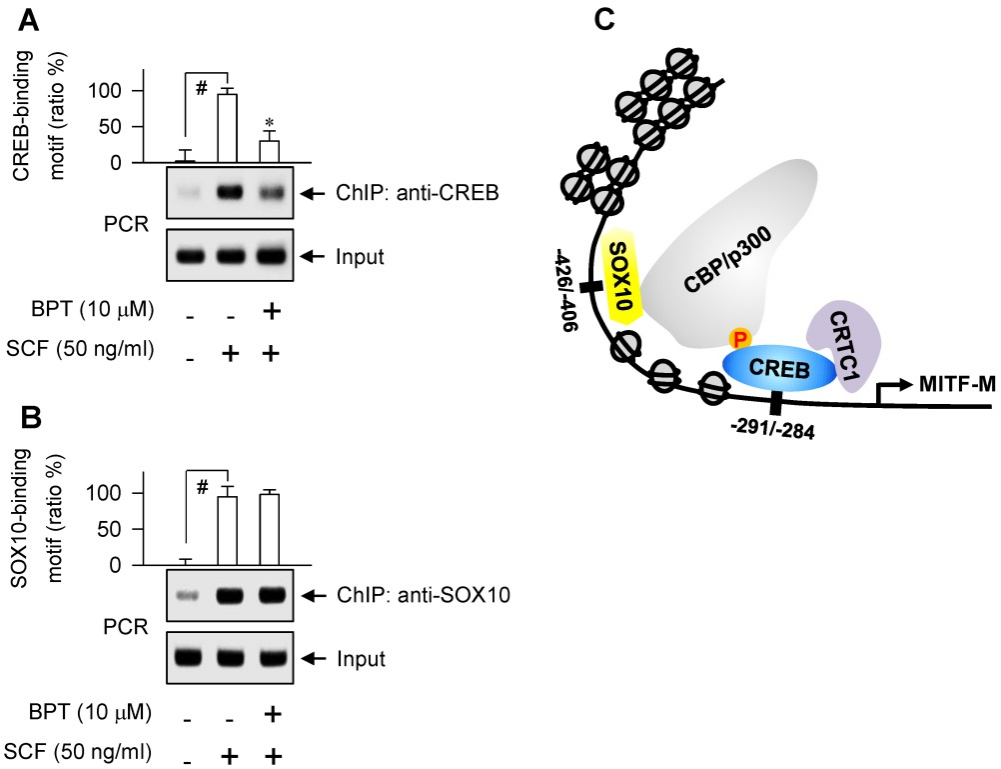
** DNA binding ability of CREB or SOX10 at the MITF-M promoter.** Chromatin immunoprecipitation assay (ChIP). B16-F0 cells were pretreated with BPT for 2 h and stimulated with SCF for 1 h in the presence of BPT. Chromatin fragments were precipitated with anti-CREB (A) or anti-SOX10 antibody (B). Input and precipitated DNAs were subjected to PCR encompassing CREB- (A) or SOX10-responsive *cis*-acting element (B) at the MITF-M promoter. Data are mean ± SEM. ^#^*P* < 0.05 vs. medium alone. *P < 0.05 vs. SCF alone. (C) A proposed model of SCF/KIT-activated transcription factors at the MITF-M promoter.

As represented in Figure S2A, proximal region of MITF-M promoter encodes a number of *cis*-acting elements 29, 30. Here, we unveiled, for the first time, which transcription factors can direct SCF/KIT-induced MITF-M expression at the promoter level. siRNA-based gene knockdown of CREB, CRTC1 or SOX10 but not β-catenin ablated the mRNA levels of MITF-M in SCF-activated B16-F0 cells (Figure 4A-D). In addition, CREB was predominantly localized in the nucleus irrespective to the absence or presence of SCF (Figure 5A), and its Ser-133 was transiently phosphorylated by SCF stimulation, whereby anti-p-CREB antibody cross-reacted with another phosphorylated cAMP-dependent transcription factor, p-ATF1 (Figure 5B). CRTC1 was translocated from the cytosol to the nucleus by SCF stimulation, following the dephosphorylation at Ser-171 (Figure 5A and C). However, SCF did not affect the protein and mRNA levels of CREB or CRTC1 (Figure 5B-D). SOX10 was localized in the nucleus after SCF-triggered gene expression (Figure 5A and D). Moreover, CREB and SOX10 occupied the *cis*-acting elements, -291/-284 and -426/-406 at the MITF-M promoter, and their specific binding was markedly elevated by SCF stimulation (Figure 6A-B). Specific phosphorylation of CREB promotes the recruitment of CBP/p300 co-activator that acetylates nucleosomal histones in chromatin disassembly, thus making DNA sequences on CREB-target genes, such as MITF-M, available to the transcription machinery 31, 32. Phosphor (p)-CRTC1 is sequestered in the cytoplasm, while the dephosphorylated CRTC1 shuttles into the nucleus and co-activates the bZIP transcription factors, such as CREB 33, 34, 35. SOX10 restricts MITF-M expression in melanocytes by cooperation with CREB/CRTC1 heteromer, known as a tissue-specific adaptation in response to cAMP, second messenger that is ubiquitous in almost all cell types 36, 37. Taken together, we propose the transcription factors that switched on the MITF-M promoter in response to SCF/KIT (Figure 6C).

BPT had no effects on the nuclear-cytoplasmic shuttling of CREB, CRTC1 or SOX10 in SCF-activated B16-F0 cells (Figure 5A), and on the dephosphorylation of CRTC1 or the induction of SOX10 (Figure 5C-D). However, BPT inhibited SCF-induced phosphorylation of CREB at Ser-133 (p-CREB) in B16-F0 and HEM cells, as did ISCK03 and imatinib (Figure 5B; Figure S2B). Topical treatment of BPT also decreased p-CREB levels in UV-B-exposed skin of HRM2 mice or brownish guinea pigs (Figure S2C-D). Furthermore, BPT inhibited SCF-enhanced occupancy of CREB but not SOX10 at the MITF-M promoter (Figure 6A-B).

### SCF/KIT-directed signaling pathway for CREB phosphorylation that linked to MITF-M expression

Either axis of the rapidly activated fibrosarcoma kinase (Raf)-ERK-RSK or p38 MAPK-MSK1 increases the transcriptional ability of CREB via phosphorylation in SCF- or UV-B-activated melanocytes 23, 38. Here, specific inhibitors of Src, MEK1/2 or RSK decreased SCF-induced phosphorylation of CREB at Ser-133 (Figure S3A), in concert with their suppressive effects on the mRNA levels of MITF-M (Figure 2). The p38 MAPK-MSK1 pathway was excluded because p38 MAPK inhibitors had no effects on SCF-induced MITF-M transcription (Figure 2, upper). Src and Ras-GTP have been shown to activate Raf activity 39. BPT decreased Ras-GTP levels in SCF-activated B16-F0 cells (Figure S3B), and sequentially inhibited the phosphorylation of c-Raf at Tyr-340 and Tyr-341 or that of ERK1/2 at Thr-202 and Tyr-204 (Figure S3C-D). The results suggest that a signaling pathway, Src-Ras-Raf-MEK1/2-ERK1/2-RSK, could link SCF/KIT to the phosphorylation (activation) of CREB for switching on the MITF-M promoter.

### Chemical inhibition of KIT activity mitigated facultative pigmentation

**Figure 7:**
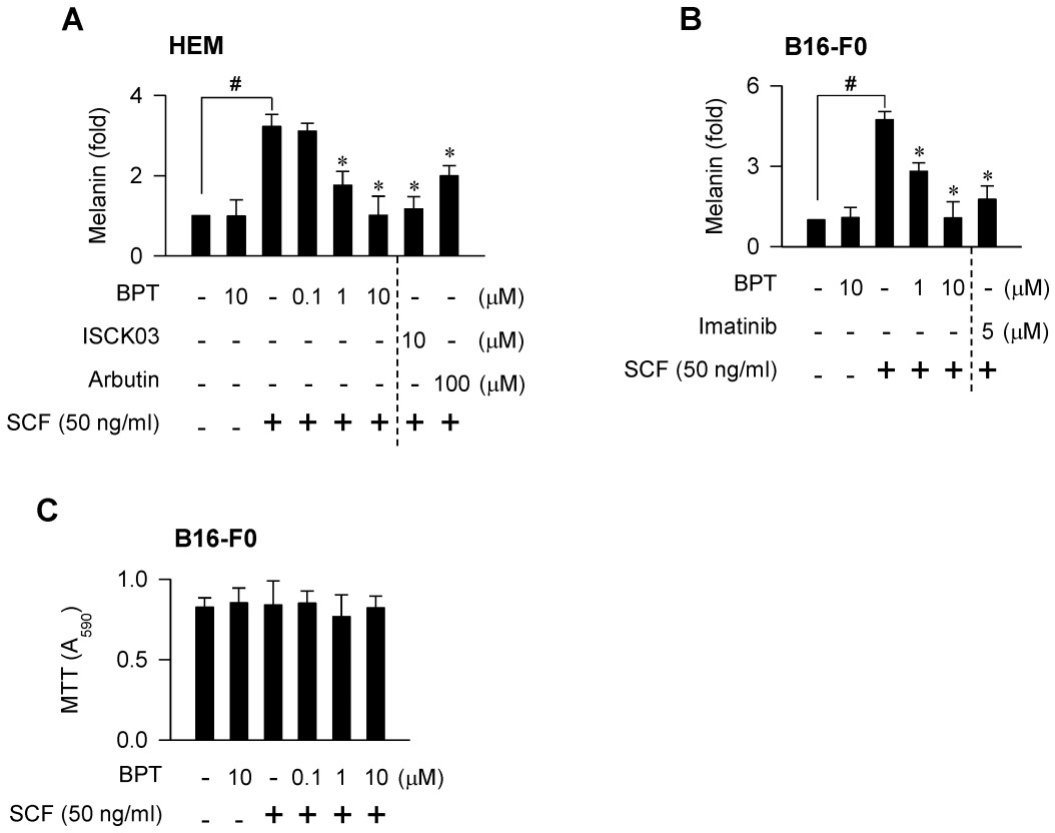
** SCF/KIT-induced pigmentation.** Melanin production in melanocyte cultures. Cells were stimulated with SCF for 96 h (A) or 72 h (B) in the presence of BPT. (C) MTT assay on the cell viability. B16-F0 cells were incubated with BPT for 72 h in the presence of SCF. Data are mean ± SEM. ^#^*P* < 0.05 vs. medium alone. *P < 0.05 vs. SCF alone.

**Figure 8:**
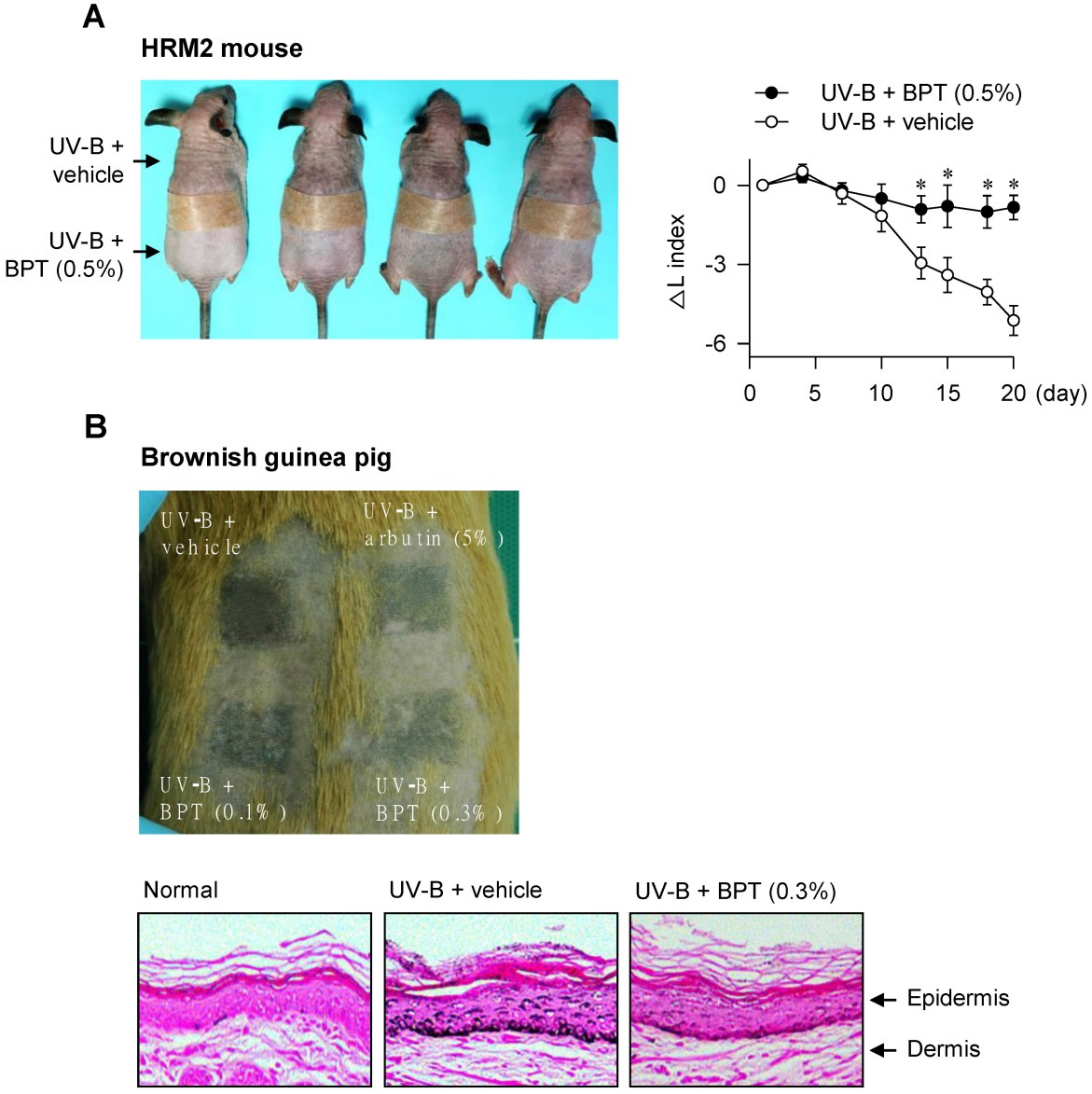
** UV-B-induced pigmentation.** The dorsal skin of HRM2 mice (A) or brownish guinea pigs (B) was irradiated with UV-B and treated topically with BPT as shown in Figure S1D-E. (A) Photograph of skin pigmentation in HRM2 mice (left). The change of lightening index (△L) in UV-B-exposed skin (right). Data are mean ± SEM. *P < 0.05 vs. UV-B plus vehicle alone. (B) Photograph of skin pigmentation in brownish guinea pigs (upper). UV-B-exposed skin that was stained with Fontana-Masson silver nitrite (lower).

BPT, as well as ISCK03 and imatinib, inhibited SCF-induced melanin production in HEM and B16-F0 cells (Figure 7A-B), as they had suppressed MITF-M expression at the promoter level (Figure 1B-C; Figure S1C). BPT had no significant effects on basal pigmentation or cell viability (Figure 7A-C). Moreover, topical treatment with BPT protected the skin from UV-B-induced hyperpigmentation in HRM2 mice or brownish guinea pigs, as did arbutin (Figure 8A-B), in which BPT had down-regulated the protein and mRNA levels of MITF-M in the skin (Figure 3A-B). Arbutin is a Korea FDA-approved skin whitener. Topical treatment with BPT restored the lightening index in UV-B-exposed and pigmented skin (Figure 8A, right), and also mitigated UV-B-induced melanin granules, evident at the epidermal/dermal border (Figure 8B, lower), where heavily pigmented melanosomes are generated in melanocytes 40.

**Figure 9:**
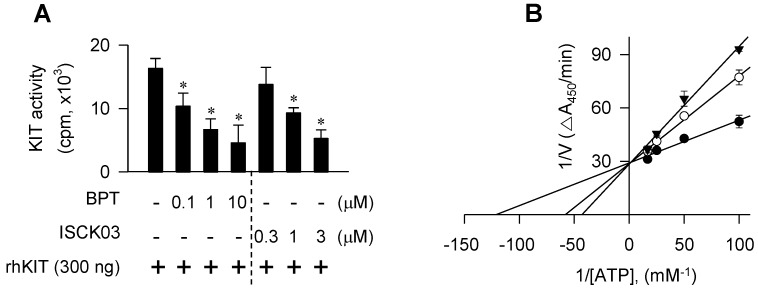
** KIT-catalyzed tyrosine kinase activity in cell-free condition.** Catalytically active rhKIT was reacted with poly(Glu,Tyr 4:1) peptide as an exogenous substrate in the presence of BPT. (A) *In vitro* kinase activity as the count per minute (cpm) by incorporating [^32^P] from [γ-^32^P]ATP to the peptide substrate. Data are mean ± SEM. *P < 0.05 vs. rhKIT alone. (B) rhKIT kinetics with varying concentrations of ATP as the absorbance change at 450 nm per minute (△A_450_/min) by ELISA. Symbols are KIT activity in the presence of 10 μM (solid triangle) or 0.1 μM of BPT (open circle) and in the absence of BPT (solid circle). Data are mean values.

**Figure 10:**
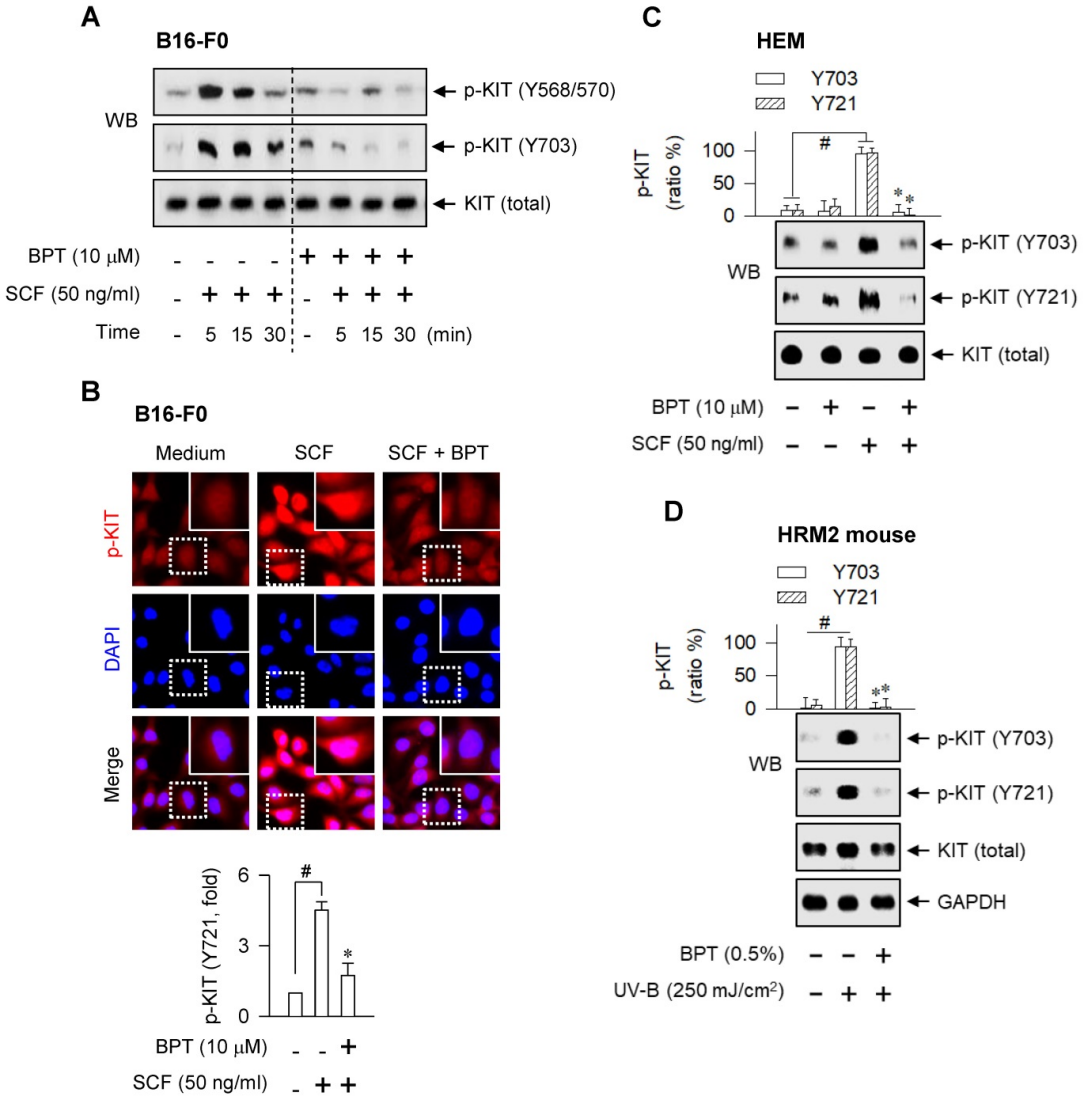
** SCF- or UV-B-induced KIT autophosphorylation.** SCF-induced KIT autophosphorylation. Cells were pretreated with BPT for 2 h and stimulated with SCF for 10 min (B, C) or indicated time points (A) in the presence of BPT. (A, C) Western blot analysis (WB). (B) Confocal fluorescence microscopy, displaying the p-KIT in red and the nuclei in blue (upper), and p-KIT levels as relative fold (lower). (D) WB on the UV-B-induced KIT autophosphorylation. The dorsal skin of HRM2 mice was irradiated with UV-B and treated topically with BPT as shown in Figure S1D. Data are mean ± SEM. ^#^*P* < 0.05 vs. medium alone (B, C) or normal skin (D). *P < 0.05 vs. SCF alone (B, C) or UV-B alone (D).

As a molecular target in the antimelanogenic activity via regulating MITF-M activity, BPT directly inhibited the tyrosine kinase activity of cell-free rhKIT, as did ISCK03 (Figure 9A). The rhKIT exhibited a *K_m_* of 8 μM and a *V_max_* of 0.034 △A_450_/min with varying concentrations of ATP (Figure 9B). Treatment with BPT increased the *K_m_* but did not alter the *V_max_* (Figure 9B), suggesting an ATP-competitive inhibition of KIT activity. However, BPT did not inhibit the kinase activity of Src, B-Raf, ERK2 or MEK1 in cell-free reactions (Figure S4A-D). We then conducted the molecular docking. BPT was arranged at the active site of KIT, consisting of Gly-598, Cys-673, Asn-797 and Asp-810, in the most favorable simulation, which overlapped with endogenous ATP bound to KIT in the crystal structure (Figure S4E). Finally, BPT inhibited SCF-induced autophosphorylation of KIT at Tyr-568, -570, -703 or -721 (p-KIT) in B16-F0 and HEM cells (Figure 10A-C). Topical BPT also decreased p-KIT levels in UV-B-exposed and pigmented skin of HRM2 mice (Figure 10D).

## Discussion

Congenital defects in any one of the proteins SCF, KIT or MITF-M lead to overlapping phenotypes, such as early loss of the melanocyte lineage 10. MITF-M is required in the maintenance of KIT expression 8, 41. Here, we elucidated that SCF/KIT could trigger the expression of MITF-M *de novo*, following the post-translational modification of pre-existing MITF-M protein. SCF/KIT stimulated the transcriptional abilities of CREB/CRTC1 heteromer and SOX10 at the MITF-M promoter. Meanwhile, chemical inhibition of KIT activity down-regulated MITF-M expression at the promoter level and abolished melanin production in SCF-activated HEM or B16-F0 cells, as well as protected the skin from UV-B-induced hyperpigmentation in HRM2 mice or brownish guinea pigs with attenuated mRNA and protein levels of MITF-M. The results suggest that MITF-M activity through gene expression could link SCF/KIT to the acquired hyperpigmentation in skin.

In the SCF/KIT-transmitted signaling pathways, Src is activated by docking to the phosphorylation sites of KIT at Tyr-568 and -570, Grb2 by binding to that at Tyr-703, and PI3K by interaction with that at Tyr-721 16, 17. Targeted mutants reveal that the phosphorylation sites of KIT at Tyr-568, -570 and -721, but not that at Tyr-703, regulate MITF-M activity via phosphorylation at the post-translation level in SCF/KIT-induced cell proliferation 42. Here, chemical inhibition of Src activity or siRNA-based gene knockdown of Grb2 had ablated SCF/KIT-induced mRNA levels of MITF-M while PI3K inhibitors had no effects, suggesting that the phosphorylation sites of KIT at Tyr-568, -570 and -703, but not that at Tyr-721, could affect MITF-M activity through gene expression.

The MAPK pathway via ERK and RSK-1 has been known to regulate MITF-M activity via phosphorylation at Ser-73 and Ser-409 in SCF/KIT-activated melanocytes 19, 20. Here, chemical inhibition of KIT, Src, MEK1/2 or RSK had decreased the phosphorylation of CREB at Ser-133 as well as suppressed the mRNA levels of MITF-M in SCF/KIT-activated HEM or B16-F0 cells. An outcome of the specific phosphorylation couples to the ubiquitin-dependent proteolysis of MITF-M 19. Here, SCF/KIT shifted the electrophoretic mobility of MITF-M via phosphorylation followed by degradation of phosphor-MITF-M in a fast kinetics, but did not affect the protein and mRNA levels of CREB. As another outcome, MITF-M and CREB with the specific phosphorylation enhance their transcriptional activity by the recruitment of CBP/p300 co-activator 21, 22, 31, but they do so at different promoters. MITF-M is a pivotal transcription factor in the expression of melanogenic genes, such as TYR, which contain the *cis*-acting M and E box at the promoter 8. Here, CREB had occupied the MITF-M promoter, and its gene knockdown with a siRNA-based approach ablated the mRNA levels of MITF-M in SCF/KIT-activated HEM or B16-F0 cells. Therefore, the same signaling pathway, Src-MEK1/2-ERK-RSK, could link SCF/KIT to MITF-M activity through dual mechanisms of gene expression and post-translational modification.

Taken together, stimulation of KIT with SCF profoundly increased the protein and mRNA levels of MITF-M in epidermal melanocytes over the course of hours, but the post-translational modification of MITF-M over the course of minutes. These alternative pathways may contribute to distinct characteristics of MITF-M activity in the context of melanogenic process. In addition, chemical inhibition of KIT activity abolished SCF-induced melanin production in epidermal melanocytes *in vitro* and protected the skin from UV-B-induced hyperpigmentation *in vivo*, in which it down-regulated MITF-M expression at the promoter level. Finally, we propose the targeting of SCF/KIT-inducible MITF-M expression as a strategy in the therapeutics for acquired pigmentary disorders such as melasma, freckles and senile lentigo.

## Materials and Methods

### Drugs, chemicals and cell lines

BPT (> 99% purity) was synthesized as previously described 26. Pharmacological agents were H-89 (Sigma-Aldrich, B1427) and Rp-cAMPS (Sigma-Aldrich, A165) as PKA inhibitors; PP2 (Sigma-Aldrich, P0042) and SU6656 (Sigma-Aldrich, S9692) as Src inhibitors; PD98059 (Sigma-Aldrich, P215) and U0126 (Sigma-Aldrich, U120) as MEK1/2 inhibitors; LY294002 (Sigma-Aldrich, L9908) and wortmannin (Sigma-Aldrich, W1628) as PI3K inhibitors; SL0101 (Merck, 559285) as an RSK inhibitor; 6BIO (Sigma-Aldrich, B1686) and SB216763 (Sigma-Aldrich, S3442) as GSK3β inhibitors; SB202190 (Sigma-Aldrich, S7067) and SB203580 (Sigma-Aldrich, S8307) as p38 MAPK inhibitors; ISCK03 (Sigma-Aldrich, I6410) and imatinib (Sigma-Aldrich, SML1027) as inhibitors of SCF/KIT signaling; and arbutin (Sigma-Aldrich, A4256) as a skin whitener. Cell lines were HEM (ThermoFisher Scientific, C1025C) and B16-F0 (ATCC, CRL-6322).

### Western blot analysis (WB)

Protein extracts were resolved on SDS-acrylamide gels (Biosesang, A2003) by electrophoresis and transferred to polyvinylidene difluoride membrane (Roche, 03010040001). Western blots were blocked with 5% non-fat milk (Becton-Dickinson, 23100) or 5% bovine serum albumin (BSA, Affymetrix, 10857) in Tris-buffered saline containing 0.05% Tween 20 (Sigma-Aldrich, P1379). After washing, the blot was reacted with primary antibody at 4℃ overnight followed by secondary antibody for 1-3 h at room temperature. Immune complexes were visualized with enhanced chemiluminescence reagent (GE Healthcare, RPN2232). This study employed primary antibodies against MITF (Abcam, ab12039), CREB (Cell Signaling, 9197), p-CREB at Ser-133 (Cell Signaling, 9198), CRTC1 (Cell Signaling, 2587), p-CRTC1 at Ser-171 (Cell Signaling, 3359), SOX10 (Santa Cruz, sc-17342), c-Raf (Cell Signaling, 9422), p-c-Raf at Tyr-340 and Tyr-341 (ThermoFisher Scientific, 44-506G), ERK1/2 (Cell Signaling, 9102), p-ERK1/2 at Thr-202 and Tyr-204 (Cell Signaling, 9101), KIT (Cell Signaling, 3074), p-KIT at Tyr-568 and Tyr-570 (Santa Cruz, sc-18076), p-KIT at Tyr-703 (Cell Signaling, 3073), p-KIT at Tyr-721 (Bioss, bs-3242R), GAPDH (Santa Cruz, sc-25778) or histone H1 (Santa Cruz, sc-8030). Secondary antibodies were horseradish peroxidase (HRP)-labeled rabbit anti-goat IgG (ThermoFisher Scientific, A27011); HRP-labeled goat anti-rabbit IgG (ThermoFisher Scientific, 31460); and HRP-labeled goat anti-mouse IgG (ThermoFisher Scientific, 31430).

### RT-PCR analysis

Total RNAs were subjected to RT-PCR analysis in the determination of mRNA levels of MITF-M, SOX10, CREB, CRTC1, β-catenin or Grb2. Nucleotide sequences of RT-PCR primers are described in Table S1. Briefly, total RNAs were reversely transcribed for 1 h at 42℃ with oligo-dT as the primer (iNtRON, 25087). Single-stranded cDNAs were subjected to 27-40 cycles of PCR using premix kit (Bioneer, K2018). One cycle consisted of denaturation for 30 s at 94℃, annealing for 30 s at 54-60℃, and DNA extension for 1 min at 72℃. RT-PCR products were resolved on agarose gels (iNtRON, 32034) by electrophoresis and stained with EcoDye (Biofact, ES301-1000).

### Luciferase reporter assay

Cells were transfected with the reporter construct, MITF-M (-2200/+95)-Luc, in combination with *Renilla* control vector, for 24 h using Lipofectamine kit (ThermoFisher Scientific, 11668). The transfected cells were stimulated with SCF for 20 h. Cell extracts were subjected to dual luciferase assay (Promega, E1910). Firefly luciferase activity, reporting the promoter activity of MITF-M, was normalized to *Renilla* activity as a reference of transfection efficiency.

### siRNA-based gene knockdown

Cells were transfected with siRNA against CREB, CRTC1, SOX10, β-catenin or Grb2 for 48 h using Lipofectamine kit. Nucleotide sequences of siRNAs are 5'-GAUUCACAGGAGUCUGUGG-3' for CREB; 5'-UGGACAGAGUAUAUCGUGA-3' for CRTC1; 5'-GGUCAAGAAGGAACAGCAG-3' for SXO10; 5'-CUGUUGGAUUGAUUCGAAA-3' for β-catenin; and 5'-GAGCCAAGGCAGAAGAAAU-3' for Grb2.

### Chromatin immunoprecipitation assay (ChIP)

Cells were incubated with 1% formaldehyde (Sigma-Aldrich, F8775) to cross-link between DNA and proteins, and sonicated to yield chromatin fragments with about 200-500 base pairs. Chromatin fragments were reacted with anti-CREB antibody (Cell Signaling, 9197) or anti-SOX10 antibody (Santa Cruz, sc-17342) at 4℃ overnight, and precipitated with protein A-sepharose bead-sheared salmon sperm slurry (Merck, 17-295) for 4 h. Input and precipitated DNAs were subjected to PCR encompassing CREB- or SOX10-responsive element at the MITF-M promoter. Nucleotide sequences of PCR primers are previously described 35. PCR products were resolved on agarose gels by electrophoresis and stained with EcoDye.

### Melanin quantification

Cells were stimulated with SCF (R&D Systems, 255-SC) for 72-96 h. Melanin pigments were disrupted in 0.85 N NaOH and 20% dimethyl sulfoxide (Sigma-Aldrich 34869) at 80^o^C, and absorbance values were measured at 405 nm.

### MTT assay

Cells were incubated with BPT for 72 h in the presence of SCF, and reacted with 0.5 mg/ml 3-(4,5-dimethylthiazol-2-yl)-2,5-diphenyltetrazolium bromide (MTT, Sigma-Aldrich, M5655) for 1 h. Formazan crystals were dissolved in 99% dimethyl sulfoxide, and absorbance values were measured at 590 nm.

### Skin pigmentation

The dorsal skin of HRM2 mice (Central Lab Animal) or brownish guinea pigs (Daehan Biolink) was irradiated with UV-B and treated topically with BPT, dissolving in the vehicle of propylene glycol: ethanol: H_2_O (5: 3: 2), according to the procedures shown in Figure S1D-E. The lightening index was measured in UV-B-exposed skin using a chromameter. Skin tissues were fixed in 4% formaldehyde, embedded in paraffin (Sigma-Aldrich, 327212), sectioned at a thickness of 5 μm, and reacted with a premix kit of Fontana-Masson silver nitrate (Scytek, FMS-1) to examine melanin granules. Protein extracts or total RNAs from skin tissues were subjected to immunoblotting or RT-PCR. IRB protocols (CBNUA-809-15-01, CBNUR-1253-19) were approved by the Animal Experimentation Ethics Committee of CBNU Institute and conducted in accordance with the Korean Ministry of Food and Drug Safety Guide for the Care and Use of Laboratory Animals.

### *In vitro* kinase assay

Catalytically active rhKIT (SignalChem, K06-12EG) or rhSrc (SignalChem, S19-18G) was reacted with 0.3 mg/ml poly(Glu,Tyr 4:1) peptide (SignalChem, P61-58) as an exogenous substrate in the presence of 5 μCi [γ-^32^P]ATP (Perkin Elmer, NEG002A) for 15 min at 30^o^C. Catalytically active rhPKC (SignalChem, P66-10G), rhB-Raf (SignalChem, B08-11G), rhMEK1 (SignalChem, M02-10G) or rhERK2 (SignalChem, M28-10G) was reacted with 0.4 mg/ml myelin basic protein (MBP, SignalChem, M42-51N) as an exogenous substrate in the presence of 5 μCi [γ-^32^P]ATP. The reaction mixtures were spotted onto P81 phosphocellulose filter, washed extensively with 0.8% H_3_PO_4_ followed by 98% acetone, and measured the radioactivity as count per minute (cpm).

Catalytic kinetics of rhKIT was determined by ELISA as described previously 43. In brief, poly(Glu,Tyr 4:1) peptide was immobilized onto microplate, and reacted with rhKIT in 10 mM MgCl_2_, 1 mM MnCl_2_, 1 mM sodium orthovanadate (Sigma-Aldrich, S6508), 20 mM HEPES (Sigma-Aldrich, H4034) and varying concentrations of ATP (Sigma-Aldrich, A50-09). After washing, the microplate was added with HRP-labeled anti-phosphotyrosine antibody (Cell Signaling, 5465), and absorbance values were measured at 450 nm in the presence of tetramethyl benzidine (Sigma-Aldrich, 860336). rhKIT-catalyzed kinetic parameters, Michaelis-Menten *K_m_* constant and maximal velocity (*V_max_*), were determined using Lineweaver-Burk plots.

### Confocal microscopy

Cells were mounted onto poly(Lys)-coated slide, fixed in 4% formaldehyde, and blocked with phosphate-buffered saline containing 1% BSA, 0.1% gelatin (Sigma-Aldrich, G7041), 0.3% Triton X-100 (Sigma-Aldrich, X100) and 0.05% Tween 20. After washing, cells were reacted with antibody against p-KIT at Tyr-721 (Bioss, bs-3242R) at 4℃ overnight followed by Alexa Fluor 594-labeled donkey anti-rabbit IgG (ThermoFisher Scientific, A21207) for 1 h in the dark, and incubated with 4,6-diamidino-2-phenylindole (DAPI, Vector, H-1200) for 3 min. The p-KIT was examined in red and the nuclei in blue under confocal fluorescence microscope.

### ELISA

Ras-GTP was quantified by ELISA according to the protocol supplied with premix kit (Active Motif, 52097). In brief, Raf-Ras binding domain was immobilized onto microplate, and reacted with cell extracts. After washing, the microplate was added with anti-H-Ras antibody followed by HRP-labeled rabbit anti-rat IgG, and chemiluminescence was measured as relative luminescence unit.

### Molecular docking

Crystal structure of human KIT was retrieved from Protein Data Bank (code no. 1PKG). Chemical structure of BPT was drawn using Chemdraw ultra, and minimized to the lowest energy using Flare kit. Molecular docking of BPT to the crystal structure of human KIT was carried out using Flare kit.

### Image quantification

The image on gels for WB, RT-PCR and ChIP was quantified using a densitometer, and are represented as relative ratio %, in which the highest intensity of band was referred to 100%.

### Statistical analysis

Results are expressed as mean ± standard deviation (n = 3, unless otherwise indicated). *P* < 0.05 was considered significantly different after statistical analysis with ANOVA followed by the Student's *t*-test.
