# Supplementary material for: Stem Cell Factor-Inducible MITF-M Expression in Therapeutics for Acquired Skin Hyperpigmentation
Source: Theranostics. 2020 Jan 1;10(1):340–52. doi: 10.7150/thno.39066 (PMC6929618; doi:10.7150/thno.39066)
Supplement: Supplementary file 1 — Supplementary figures and tables. [file thnov10p0340s1.pdf]

Supplementary Material

Stem Cell Factor-Inducible MITF-M Expression in Therapeutics for Acquired Skin Hyperpigmentation

Cheong-Yong Yun, Eunmiri Roh, Song-Hee Kim, Jinhe Han, Jiyeon Lee, Da-Eun Jung, Ga Hyeon Kim, Sang-Hun Jung, Won-Jea Cho, Sang-Bae Han, Youngsoo Kim

---

Contents

Figure S1. MITF-M expression in SCF-activated B16-F0 cells

Figure S2. Effect of BPT on SCF/KIT- or UV-B-induced phosphorylation of CREB

Figure S3. SCF/KIT-directed signaling pathway

Figure S4. Effect of BPT on the kinase activity of cell-free Src, B-Raf, ERK2 or MEK1

Table S1. Nucleotide sequence of RT-PCR primer

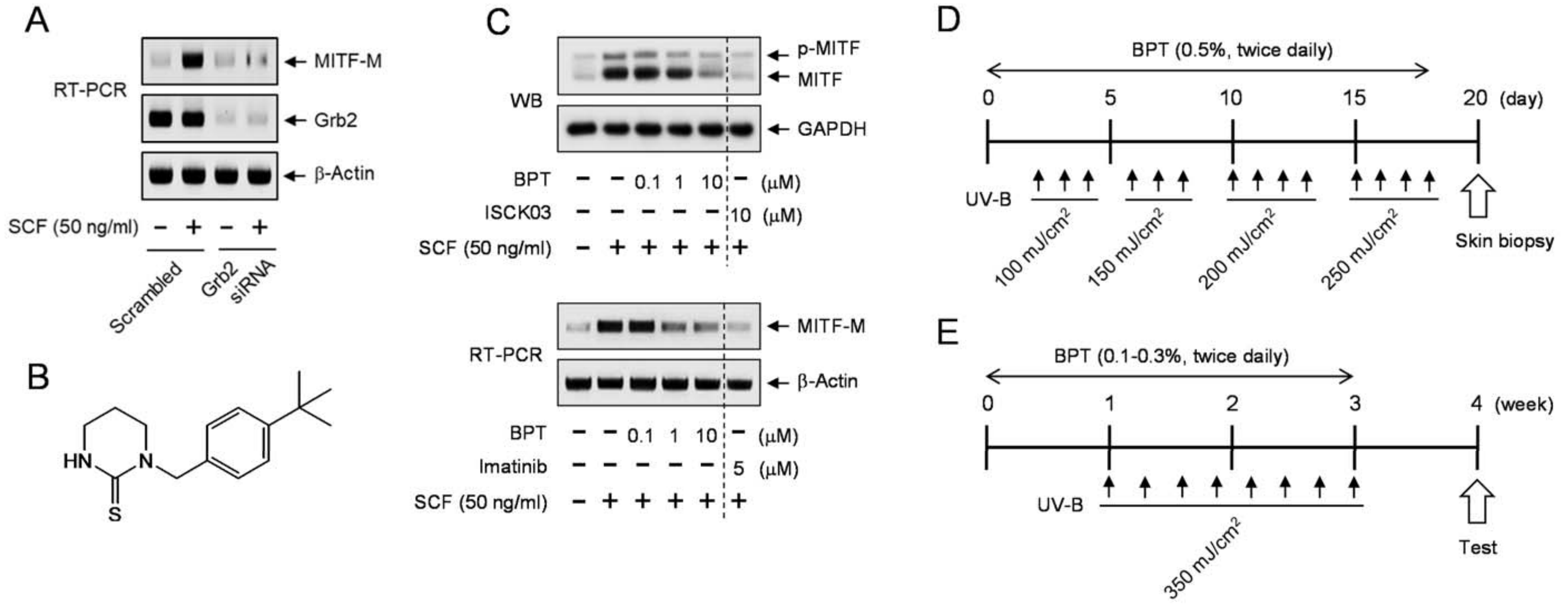

Figure S1. MITF-M expression in SCF-activated B16-F0 cells.

(A) RT-PCR analysis of MITF-M. B16-F0 cells were transfected with siRNA against Grb2 for 48 h and stimulated with SCF for 2 h. (B) Chemical structure of BPT. (C) Western blot analysis (WB) and RT-PCR analysis of MITF-M. B16-F0 cells were pretreated with BPT for 2 h and stimulated with SCF for 4 h (WB) or 2 h (RT-PCR) in the presence of BPT. (D, E) Experimental protocol of UV-B-induced skin hyperpigmentation. The dorsal skin of HRM2 mice (D) or brownish guinea pigs (E) was topically treated with BPT in a twice-daily regimen, and irradiated with UV-B lamp (wavelength 290-315 nm) at the time points indicated by an arrow.

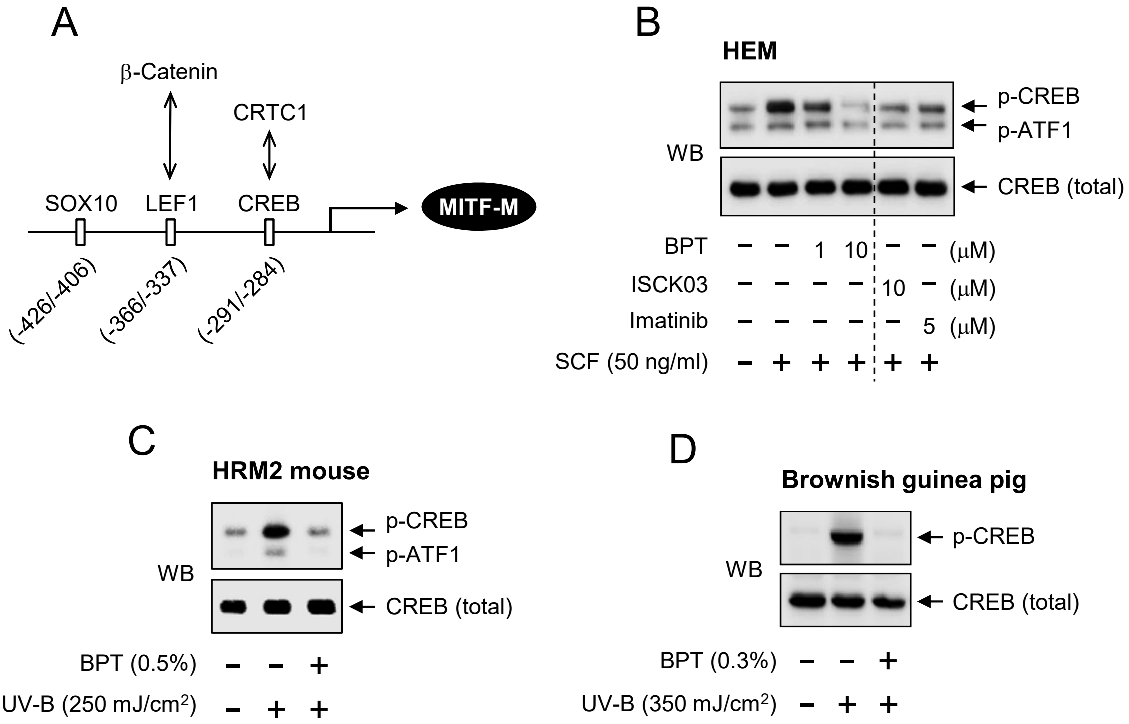

Figure S2. Effect of BPT on SCF/KIT- or UV-B-induced phosphorylation of CREB.

(A) Proximal region at the MITF-M promoter. The CREB-, LEF1- or SOX10-responsive *cis*-acting elements are located at -291/-284, -366/-337 or -426/-406 of the MITF-M promoter in mouse. CRTC1 and β-catenin co-activate CREB and LEF1, respectively. (B) Western blot analysis (WB) on the SCF-induced CREB phosphorylation. HEM cells were pretreated with BPT for 2 h and stimulated with SCF for 30 min in the presence of BPT. (C, D) WB on the UV-B-induced CREB phosphorylation. The dorsal skin of HRM2 mice or brownish guinea pigs was irradiated with UV-B and treated topically with BPT as shown in Figure S1D-E.

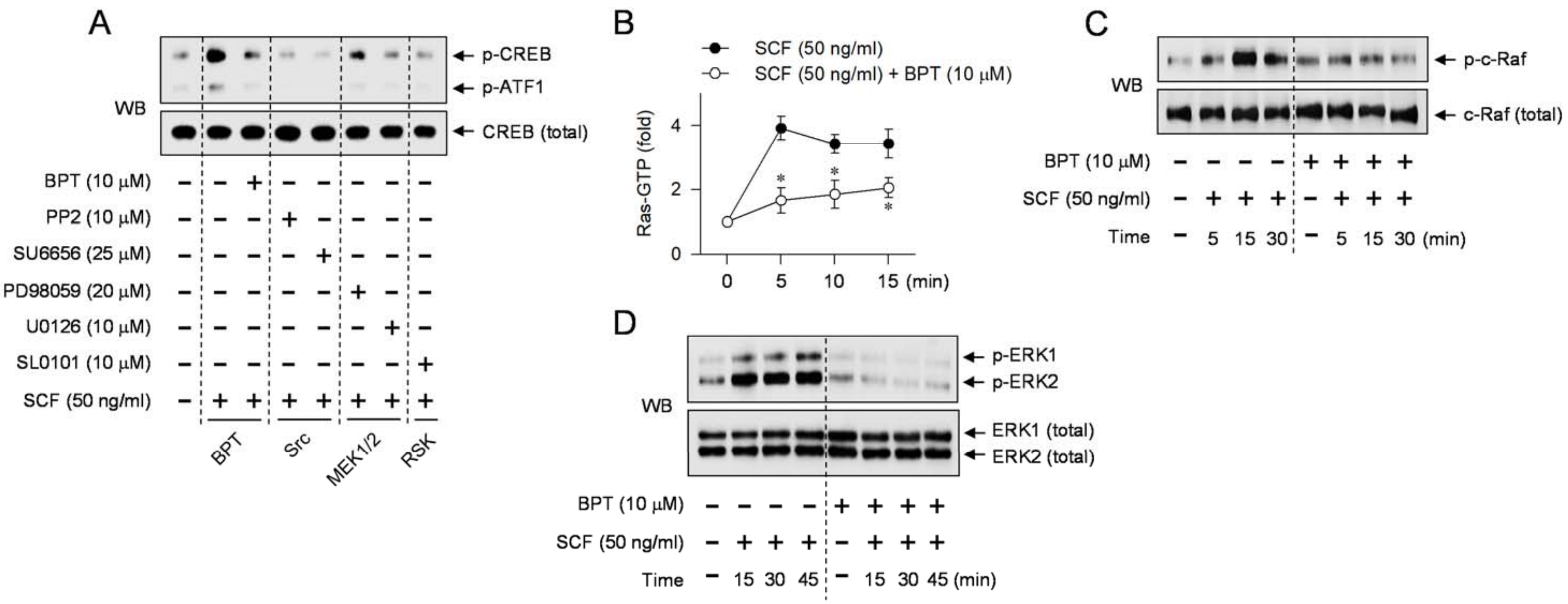

Figure S3. SCF/KIT-directed signaling pathway.

(A) Western blot analysis (WB) on the phosphorylation of CREB. B16-F0 cells were pretreated with kinase inhibitor for 2 h and stimulated with SCF for 30 min in the presence of kinase inhibitor. (B) ELISA of Ras-GTP. B16-F0 cells were pretreated with BPT for 2 h and stimulated with SCF for indicated time points in the presence of BPT. Data are mean ± SEM. *$P < 0.05$ vs. SCF alone. (C, D) WB on the phosphorylation of c-Raf (C) or ERK1/2 (D). B16-F0 cells were pretreated with BPT for 2 h and stimulated SCF for indicated time points in the presence of BPT.

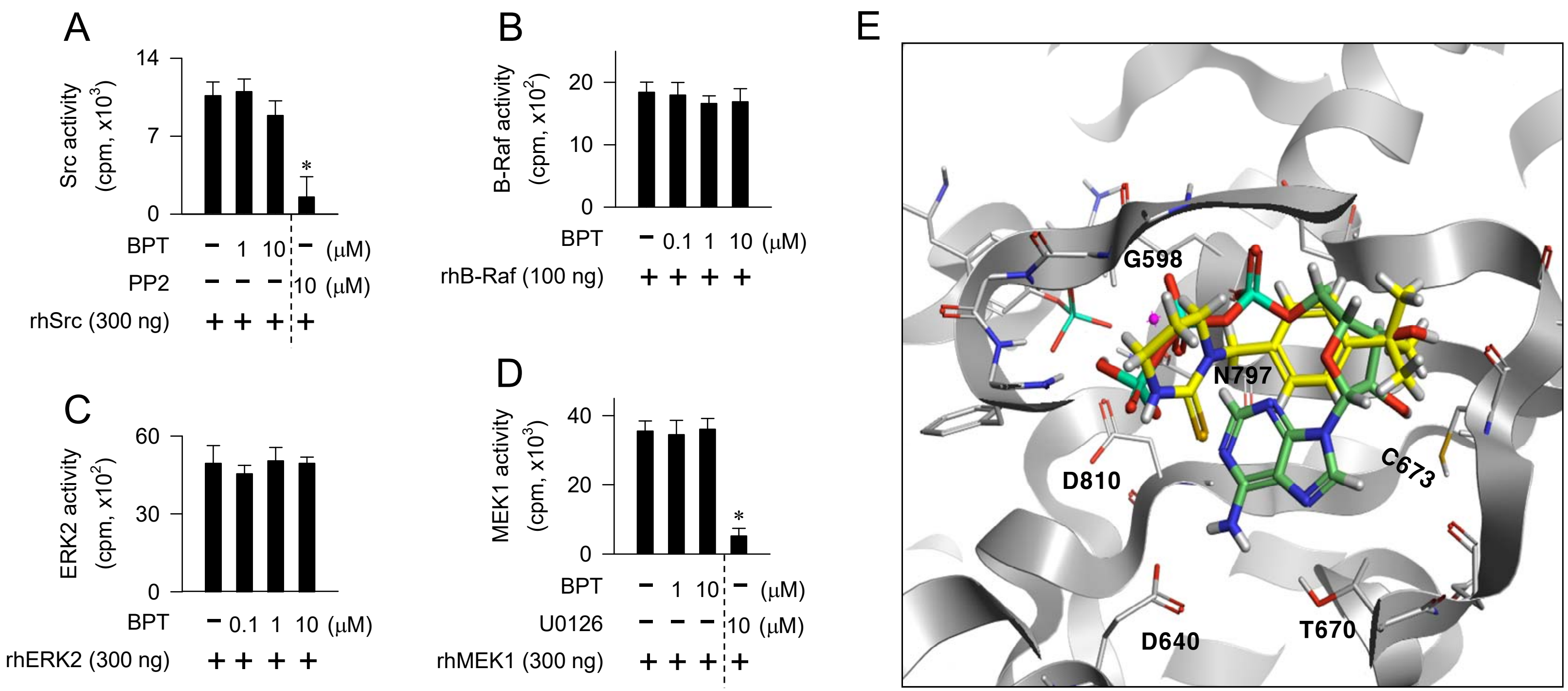

Figure S4. Effect of BPT on the kinase activity of cell-free Src, B-Raf, ERK2 or MEK1.

(A-D) *In vitro* kinase assays. Cell-free rhSrc (A), rhB-Raf (B), rhERK2 (C) or rhMEK1 (D) was reacted with poly(Glu,Tyr 4:1) peptide (A) or MBP (B-D) as exogenous substrate in the presence of BPT. The kinase activity was measured as count per minute (cpm) by incorporating [^{32}P] from [γ-^{32}P]ATP to the substrate. Data are mean ± SEM. *P < 0.05 vs. rhSrc or rhMEK1 alone. (E) Molecular docking of BPT to the crystal structure of KIT. Docking arrangement of BPT to the active site of KIT was carried out in the most favorable simulation, and superimposed onto the endogenous ATP bound to KIT in the crystal structure. BPT is indicated in yellow, ATP in green and KIT in gray.

Table S1. Nucleotide sequence of RT-PCR primer.

Target	Nucleotide sequence (amplicon)	
CREB (B16-F0)	Forward	5’-AAGCTGAAAGTCAACAAATGACAGTT-3’
	Reverse	5’-TGGACTGTCTGCCCATTGG-3’ (139 bp)
CRTC1 (B16-F0)	Forward	5’-TCCCCAACATCATCCTCAC-3’
	Reverse	5’-GGTCAATCTTCAGCTCGTC-3’ (138 bp)
Grb2 (B16-F0)	Forward	5’-CCATCGCCAAATATGACTTCAAA-3'
	Reverse	5’-TTTCTTCTGCCTTGGCTCTG-3' (210 bp)
MITF-M (B16-F0)	Forward	5’-CTAGAATACAGTCACTACCAG-3’
	Reverse	5’-CCATCAAGCCCAAAATTTCTT-3’ (404 bp)
MITF-M (B16-F0, HRM2)	Forward	5’-TACAGTCACTACCAGGTGCAG-3’
	Reverse	5’-CCATCAAGCCCAAAATTTCTT-3’ (397 bp)
MITF-M (HEM)	Forward	5'-TCTACCGTCTCTCACTGGATTGG-3'
	Reverse	5'-GCTTTACCTGCTGCCGTTGG-3’ (142 bp)
SOX10 (B16-F0)	Forward	5’-CAGACTGGAGGAGAGGTCGG-3’
	Reverse	5’-GGTCTTGTTCCTCGGCCATG-3’ (122 bp)
β-Actin (B16-F0, HRM2)	Forward	5’-TGGAATCCTGTGGCATCCATGAAAC-3’
	Reverse	5’-TAAAACGCAGCTCAGTAACAGTCCG-3’ (349 bp)
β-Actin (HEM)	Forward	5'-GAGACCTTCAACACCCCAGCC-3'
	Reverse	5'-GGCCATCTCTTGCTCGAAGTC-3‘ (312 bp)

Abbreviation: bp, base pairs.
